# Factors Influencing the Live Birth Rate Following Fresh Embryo Transfer Cycles in Infertile Women After Endometrioma Cystectomy

**DOI:** 10.3389/fmed.2021.622087

**Published:** 2021-02-25

**Authors:** Wei Liu, Tongye Sha, Yuzhen Huang, Zizhen Guo, Lei Yan, Jinlong Ma

**Affiliations:** ^1^School of Medicine, Cheeloo College of Medicine, Shandong University, Jinan, China; ^2^Center for Reproductive Medicine, Cheeloo College of Medicine, Hospital Affiliated to Shandong University, Shandong University, Jinan, China; ^3^Department of Obstetrics and Gynecology, Shanxi Bethune Hospital, Shanxi Medical University, Taiyuan, China; ^4^Department of Obstetrics, Rizhao Hospital of Traditional Chinese Medicine, Rizhao, China

**Keywords:** endometrioma cystectomy, live birth rate, ovarian function, *in vitro* fertilization, case-control study

## Abstract

**Background:** Reproductive outcomes after fresh *in vitro* fertilization/intracytoplasmic sperm injection–embryo transfer (IVF/ICSI–ET) cycles are diverse in infertile women with a history of ovarian cystectomy for endometriomas. We aimed to develop a logistic regression model based on patients' characteristics including number of embryos transferred and stimulation protocols to predict the live birth rate in fresh IVF/ICSI–ET cycles for such patients.

**Methods:** We recruited 513 infertile women with a history of ovarian cystectomy for endometriomas who underwent their first fresh ET with different stimulation protocols following IVF/ICSI cycles in our unit from January 2014 to December 2018. One or two embryo are implanted. Clinical and laboratory parameters potentially affecting the live birth rate following fresh ET cycles were analyzed. Univariable and multivariable analyses were performed to assess the relationship between predictive factors and live birth rate.

**Results:** The overall live birth rate was 240/513 (46.8%). Multivariable modified Poisson regression models showed that two factors were significantly lowers the probability of live birth: female age ≥ 5 years (aOR 0.603; 95% CI 0.389–0.933; *P* = 0.023); BMI range 21–24.99 kg/m^2^ compared with BMI <21 kg/m^2^ (aOR 0.572; 95% CI 0.372–0.881, *P* = 0.011). And two factors significantly increased the probability of live birth: AFC >7 (aOR 1.591; 95% CI 1.075–2.353; *P* = 0.020); two embryos transferred (aOR 1.607; 95% CI 1.089–2.372; *P* = 0.017).

**Conclusions:** For these infertile women who had undergone ovarian cystectomy for endometriosis, female age <35 years, AFC > 7, and two embryos transferred might achieve better clinical fresh IVF/ICSI–ET outcomes. BMI <21 kg/m^2^ or ≥25 kg/m^2^ might also have positive effects on the live birth rate, but different ovarian stimulation protocols had no significant effects. However, a larger sample size may be needed for further study.

## Introduction

Endometriosis is defined as the appearance of endometrial glands and stroma in ectopic locations, including ectopic pelvic peritoneum, ovary, and rectovaginal septum. It affects nearly 10% of women of childbearing age and 30–50% of women with chronic pelvic pain and/or infertility (1). Ovarian endometriotic cysts, also known as endometriomas, are so-called “chocolate cysts,” which are usually associated with infertility and increase the risk of endometriosis-related ovarian cancer (2). Laparoscopic surgery is currently considered to be the gold standard for diagnosing infertility related to endometriosis, removing lesions, improving pelvic structure, evaluating fallopian tube and ovarian conditions, and improving the reproductive status of such patients (3, 4). Endometriosis in infertile patients has always been a challenge for clinicians. Such patients usually present with infertility and are eager to meet their reproductive needs through assisted reproductive technology (ART). The most common adjuvant therapies are *in vitro* fertilization (IVF) and intracytoplasmic sperm injection (ICSI) and embryo transfer (ET) (5).

Should endometriomas be excised before ART? Current guidelines are unclear regarding the benefits of prior endometrioma resection. Some evidence indicates that ovarian surgery simply decreases the amount of viable ovarian tissue (6, 7). Moreover, surgical manipulation might reduce the ovarian reserve, impede ovarian response, and possibly decrease the ART success rate (8–10). A systematic meta review of 13 studies has shown that surgical and expectant management of endometriomas prior to IVF yielded similar clinical pregnancy and live birth rates, antral follicle count (AFC), mature oocyte numbers, and miscarriage rates (11). Thus, endometriomas *per se* seems to be the main reason for the long-term decline in fertility of patients, and the contribution of surgery is little. Routine cystectomy for endometriomas prior to IVF might not be necessary (11, 12). However, some studies have suggested that surgery can be beneficial. Conservative management of endometriomas in patients undergoing IVF is associated with poorer response to gonadotropin stimulation (13, 14), lower spontaneous ovulation rates (15), and worse oocyte quality (16). Large endometriomas can increase the difficulty of oocyte retrieval, as well as the risk of infection (17, 18). In addition, the proposed indications for resection of a suspected endometrioma prior to ART (19) are: (1) Rapid growth; (2) Present sonographic feature of malignancy; (3) Monolateral disease; (4) Pain symptoms; (5) Intact ovarian reserve; and (6) None previous interventions for endometriosis.

For patients with endometriosis who opt for surgical treatment because of oversized endometriomas affecting oocyte retrieval or chronic pelvic pain or suspected malignant tumor, what factors influence the live birth rate in endometriosis? Which management is more likely to succeed? Some clinicians have found that women with endometriosis and previous cystectomy may respond poorly to gonadotropin stimulation, which may result in less oocyte acquisition and poor pregnancy outcomes. Endometriosis reduces the cumulative live birth rate by reducing the number of embryos that can be transferred rather than their quality (20). A retrospective study comparing three controlled ovarian stimulation protocols for IVF–ET in patients after cystectomy for endometriomas found that a prolonged gonadotropin releasing hormone (GnRH) agonist regimen may achieve better results clinical IVF-ET results, but no significant difference from other groups (21). It has been suggested that age <35 years, infertility duration <5 years, secondary infertility, endometriosis fertility index (EFI) score and the use of ART were the predictive factors for postoperative pregnancy success (22). Advanced endometriosis has negative effect on the cumulative clinical pregnancy rate per oocyte retrieval cycle, and the AFC is an independent predictor of this (23). A recent study enrolling 485 patients with different stages of endometriosis undergoing their first IVF cycle concluded that female age, bilateral AFC and the number of embryos transferred were significant independent predictors of live birth (24). In fact, a significant proportion of women with endometriosis-related infertility choose to have surgery not to increase their chances of pregnancy, but to improve their quality of life. The question what we focused on is not whether pre-ART surgery is better than direct ART, but what are their chances of having a live birth and finding ways to increase them for patients after endometrioma cystectomy?

Therefore, the purpose of this study was to identify the predictors affecting the clinical outcomes of in fresh IVF/ICSI–ET cycles among infertile women who had undergone ovarian cystectomy for endometriomas. And to investigate whether different stimulation protocols influence the live birth rate. This may help fertility specialists and their patients to determine the expectations of appropriate treatment strategies for ovarian endometrioma before starting assisted reproductive treatment.

## Methods

### Study Design and Population

This was a retrospective study using the records of infertile women with a history of ovarian cystectomy for endometriomas who underwent the first fresh ET cycle following IVF/ICSI treatment at the Reproductive Hospital Affiliated to Shandong University from January 2014 to December 2018. This original study was approved by the Institutional Review Board of the School of Medicine, Shandong University (No. 2019-1-14). In all, the records of 513 patients were collected, of whom 240 had achieved a live birth. Both laparoscopic and open techniques were used for cystectomy for endometriomas. The inclusion criteria included: (1) ovulatory women aged ≤ 40 years; (2) women with a history of surgery for unilateral or bilateral ovarian endometrioma(s); (3) women undergoing the first cycle of IVF/ICSI treatment and receiving a fresh ET; and (4) ovarian stimulation protocols including long/prolonged/short GnRH agonist protocols and a GnRH antagonist protocol. The exclusion criteria were the presence of the following: (1) uterine malformations; (2) untreated submucosal uterine fibroids or multiple uterine fibroids; (3) adenomyosis; (4) male or female chromosomal abnormalities; and (5) patients who had undergone cyst aspiration or oophorectomy.

### Procedures

All participants were monitored and managed according to the hospital's clinical protocols. Doctors chose different ovarian stimulation protocols on the basis of long-term clinical experience, combined with the patient's age, ovarian function and other individual conditions. There were four stimulation regimens used: a long GnRH agonist protocol (186 cycles, 36.3%); a prolonged GnRH agonist protocol (105 cycles, 20.5%); a short GnRH agonist protocol (169 cycles, 33.9%); or a GnRH antagonist protocol (53 cycles, 10.3%) (25). Doctors opt different ovarian stimulation protocols on accounted of woman's age, ovarian function, clinical experience and other different personal factors. Human chorionic gonadotropin (hCG 4,000–8,000 IU) was administered when the largest follicle reached 18 mm or at least 2 follicles reached 17 mm in diameter. Oocyte retrieval was conducted by transvaginal ultrasound-guided follicular aspiration 34–36 h after the hCG trigger. After follicular aspiration, conventional IVF or ICSI was performed according to the male partner's semen quality. Vaginal progesterone gel use continued until the fetal heartbeat was heard, and oral dydrogesterone was stopped at 10 weeks of gestation. Individually cultured embryos were evaluated on the basis of the number of blastomeres, blastomere size, fragmentation rate, and presence of multinucleated blastomeres. Cleavage or blastocyst stage embryos were ultimately chosen to be transferred at 3 or 5 days after fertilization. Pregnancies were determined using β-hCG levels in blood tests performed 14 days after embryo transfer. Clinical pregnancies were confirmed by the presence of a gestational sac on vaginal ultrasonography during the fifth week. Miscarriage was defined as a pregnancy lost before 12 full weeks of gestation. Live birth was defined as the delivery of any viable infant at ≥28 weeks of gestation. All pregnancies were included in the follow-up assessment until delivery.

### Statistical Analysis

Statistical analysis was performed using IBM SPSS Statistics (v. 25.0; IBM Corp, Armonk, NY, USA). Quantitative data with normal distribution are expressed as the mean ± standard deviation (SD) and were compared with independent-samples Student's *t*-tests. Quantitative data with non-normal distribution are expressed as medians and (range) compared with the Wilcoxon signed-rank test. Categorical data are represented as frequencies and percentages; variables in these measures were compared between live birth and covariates using Pearson's chi-squared tests. A *P* < 0.05 was assumed to be significant. Variables included in logistic regression models were selected on the basis of those shown to have a relationship with the main outcomes as characterized by *P* < 0.10. Results are presented as odds ratio (OR) and confidence interval (CI). Backward stepwise selection was performed to determine independent covariates. A *P*-value of 0.05 was considered significant. Because the study was retrospective, patients were not randomly assigned to groups and there were no missing data to be considered. Receiver operating characteristic (ROC) curve analysis was performed to evaluate the performance of the live birth rate prediction model. Forest plot were performed using Graphpad Prism (v. 7.0; GraphPadSoftware, USA).

## Results

In this study, the records of 513 IVF/ICSI procedures in patients who had undergone ovarian cystectomy for endometriomas met the criteria and were included in this analysis. There were 284 pregnancies (55.4%), of which 38 (7.4%) were followed by miscarriages and 6 (1.2%) were ectopic. There were 240 live births (46.8%). Patient characteristics are summarized in Table 1. The 2 groups were similar in terms of the type of infertility, surgical side of cystectomy for removing endometriomas, interval between cystectomy and ET, duration of infertility, and basal FSH and E_2_ levels. In the non-live birth group, 155 women are primary infertility and 118 women are secondary infertility. In the live birth group, 137 women are primary infertility and 103 women are secondary infertility. Women with a successful ART cycle were younger (<35 years, *P* = 0.005). The live birth rate for woman with a BMI range of 21–24.99 kg/m^2^ was lower than in women with BMI <21 kg/m^2^ and BMI ≥ 25 kg/m^2^ (*P* = 0.003). There were no significant differences in the basal luteinizing hormone (LH) level between the groups without and with a live birth (4.76 ± 1.71 IU/L vs. 5.07 ± 2.13 IU/L, respectively; *P* = 0.067).

**Table 1:** Demographic and main treatment characteristics for patients after endometrioma cystectomy.

**Characteristic**	**Non-live birth**	**Live birth**	***P*-value**
	**(*n* = 273)**	**(*n* = 240)**	
**Age (years)**			
<35	190 (69.6%)	193 (80.4%)	0.005^a^
≥35	83 (30.4%)	47 (19.6%)	
Mean ± SD	31.81 ± 3.97	31.16 ± 3.62	0.055^a^
**Body mass index (kg/m**^**2**^**)**			
<21	70 (25.6%)	83 (34.6%)	0.003^b^
21–24.99	142 (52.0%)	89 (37.1%)	
≥25	61 (22.4%)	68 (28.3%)	
Mean ± SD	23.10 ± 3.21	22.92 ± 3.41	0.547^a^
**Type of infertility**			
Primary infertility, *n* (%)	155 (56.8%)	137 (57.1%)	0.944^b^
Secondary infertility, *n* (%)	118 (43.2%)	103 (42.9%)	
**Surgical side of endometrioma cystectomy**			
Unilateral endometrioma cystectomy, *n* (%)	182 (66.7%)	166 (69.2%)	0.545^b^
Bilateral endometrioma cystectomy, *n* (%)	91 (33.3%)	74 (30.8%)	
**Time of surgeries**			0.413^b^
Primary surgery	256 (93.8%)	229 (95.4%)	
Recurrent surgery	17 (6.2%)	11 (4.6%)	
Interval between cystectomy and embryo transfer (month)-Media (range)	27 (2–232)	24.5 (1–156)	0.406^c^
Duration of infertility (years)-Media (range)	3.82 ± 2.71	3.79 ± 2.46	0.675^a^
Basal FSH (IU/L)-Mean ± SD	7.83 ± 2.63	7.68 ± 2.71	0.529^a^
Basal LH (IU/L)-Mean ± SD	4.76 ± 1.71	5.07 ± 2.13	0.067^a^
Basal E2 (pg/ml)-Mean ± SD	43.83 ± 23.76	43.19 ± 28.26	0.780^a^

a *Independent-samples Student's t-tests*.

b *Pearson's chi-squared tests*.

c *Wilcoxon signed-rank test*.

*Results are significantly different if P < 0.05*.

*FSH, follicle stimulating hormone; LH, luteinizing hormone; E2 estradiol*.

### Controlled Ovarian Stimulation Parameters and IVF/ICSI Cycle Characteristics

The embryological and clinical factors that might have affected the live birth rate after ET are shown in Table 2. Compared with the long GnRH agonist protocol, the live birth rate for woman treated with the short GnRH agonist protocol was lower, while for woman subjected to the prolonged GnRH agonist and the GnRH antagonist protocols the birth rates were not statistically different from those subjected to the long GnRH agonist protocol (*P* = 0.013). We found that the live birth rate for women with an AFC > 7 was higher than in those with AFCs ≤ 7 (*P* = 0.004). The live birth rates were significantly higher with a start-up gonadotropin dosage <200 IU (*P* = 0.029), with cleavage stage embryos transferred (*P* = 0.032), and with two embryos transferred (*P* = 0.004). There were no statistically significant differences in live birth rates in terms of oocyte fertilization method, total gonadotropin dosage, endometrial thickness on the hCG trigger day, E2 level on the hCG trigger day, number of oocytes obtained, and the numbers of top-quality embryos transferred. There were only 28 patients with BMI <18.5 kg/m^2^ and 18 with BMI ≥30 kg/m^2^, and most of the women were concentrated in the range of 18.5–30 kg/m^2^. Thus, we could divide them into groups according to WHO criteria (<18.5 kg/m^2^, underweight; 18.5–24.99 kg/m^2^, normal or healthy weight; 25.0–29.9 kg/m^2^, overweight; ≥30 kg/m^2^, obese), but also divide each group into subgroups for analysis. Therefore, we analyzed the live birth rate for every unit of BMI, and observed a significant decrease in the live birth rate for women with a BMI of 21–24.99 kg/m^2^, so we divided the BMI into the following 3 groups: <21 kg/m^2^, 21–24.99 kg/m^2^, and ≥25 kg/m^2^. The dramatic decline in fertility for women aged ≥35 years is widely accepted as dogma in ART clinics. So we divide by age into ≥35 years and <35 years groups.We defined poor ovarian reserve as an AFC of ≤ 7 and normal ovarian reserve as an AFC of >7. In the original data, some values for the E_2_ level on the hCG trigger day were recorded as continuous variables, while some were recorded as >3,000 pg/mL, so we divided them into 3 groups: <2,000 pg/mL, 2,000–3,000 pg/mL, and ≥3,000 pg/mL. The cut-off values of gonadotropin treatment duration, start-up gonadotropin dosage, total gonadotropin dosage, and endometrial thickness on the hCG trigger day were all located near the mean or median, where they had the greatest statistical effects.

**Table 2:** Controlled ovarian stimulation parameters and IVF/ICSI cyles characteristics in patients after endometrioma cystectomy.

**Characteristic**	**Non-live birth**	**Live birth**	***P*-value**
	**(*n* = 273)**	**(*n* = 240)**	
**Ovarian stimulation protocol**			
GnRH agonist long protocol, *n* (%)	83 (30.4%)	103 (42.9%)	0.013^b^
GnRH agonist prolonged protocol, *n* (%)	55 (20.1%)	50 (20.8%)	
GnRH agonist short protocol, *n* (%)	104 (38.1%)	65 (7.1%)	
**Oocyte fertilization method**			
IVF, *n* (%)	242 (88.6%)	202 (84.2%)	0.138^b^
ICSI, *n* (%)	31 (11.4%)	38 (15.8%)	
**Gonadotropin duration (days)**			
≤ 11	176 (64.5%)	132 (55.0%)	0.029^b^
>11	97 (35.5%)	108 (45.0%)	
Mean ± SD	10.90 ± 2.31	11.39 ± 2.36	
**Start-up gonadotropin dosage (IU)**			
<200 IU	117 (42.9%)	126 (52.5%)	0.029^b^
≥200 IU	156 (57.1%)	114 (47.5%)	
Mean ± SD	204.21 ± 68.15	190.83 ± 63.77	
**Total gonadotropin dosage (IU)**			
<2,000 IU	128 (46.9%)	106 (44.2%)	0.537^b^
≥2,000 IU	145 (53.1%)	134 (55.8%)	
Media (range)	2025.00 (637.50–6750.00)	2150.00 (500.00–7500.00)	
**Endometrial thickness on HCG trigger day (cm)**			
<1.1 cm	115 (42.1%)	82 (36.8%)	0.064^b^
≥1.1 cm	158 (57.9%)	158 (63.2%)	
Mean ± SD	1.12 ± 0.23	1.15 ± 0.20	
**Ostradiol level on HCG trigger day (pg/ml)**			
<2,000	102 (37.4%)	100 (41.7%)	0.186^b^
2,000–3,000	92 (33.7%)	63 (26.2%)	
≥3,000	79 (28.9%)	77 (32.1%)	
**Antral follicle count (AFC)**			
≤ 7	108 (39.6%)	66 (27.5%)	0.004^b^
>7	165 (60.4%)	174 (72.5%)	
Mean ± SD	9.24 ± 4.44	10.35 ± 4.99	
Number of ocytes≥14 mm on HCG trigger day–Mean ± SD	7.42 ± 3.83	7.42 ± 3.65	1.000^a^
Number of oocytes obtained–Mean ± SD	7.43 ± 4.20	7.82 ± 4.00	0.280^a^
**Stage of embryos transferred**			
Cleavage transfer; *n* (%)	200 (73.3%)	195 (81.2%)	0.032^b^
Blastocyst transfer; *n* (%)	73 (26.7%)	45 (18.8%)	
**Number of top quality embryos transferred-*****n*** **(%)**			
0	158 (57.9%)	137 (57.0%)	0.217^b^
1	50 (18.3%)	33 (13.8%)	
2	65 (23.8%)	70 (29.2%)	
**Number of embryos transferred;** ***n*** **(%)**			
1	106 (38.8%)	64 (26.7%)	0.004^b^
2	167 (61.2%)	176 (73.3%)	

a *Independent-samples Student's t-tests*.

b *Pearson's chi-squared tests*.

*Results are significantly different if P < 0.05*.

*GnRH, gonadotrophin-releasing hormone; HCG, human chorionic gonadotropin*.

### Logistic Regression of Factors Related to Live Birth in IVF/ICSI Cycles

**Figure 1:**
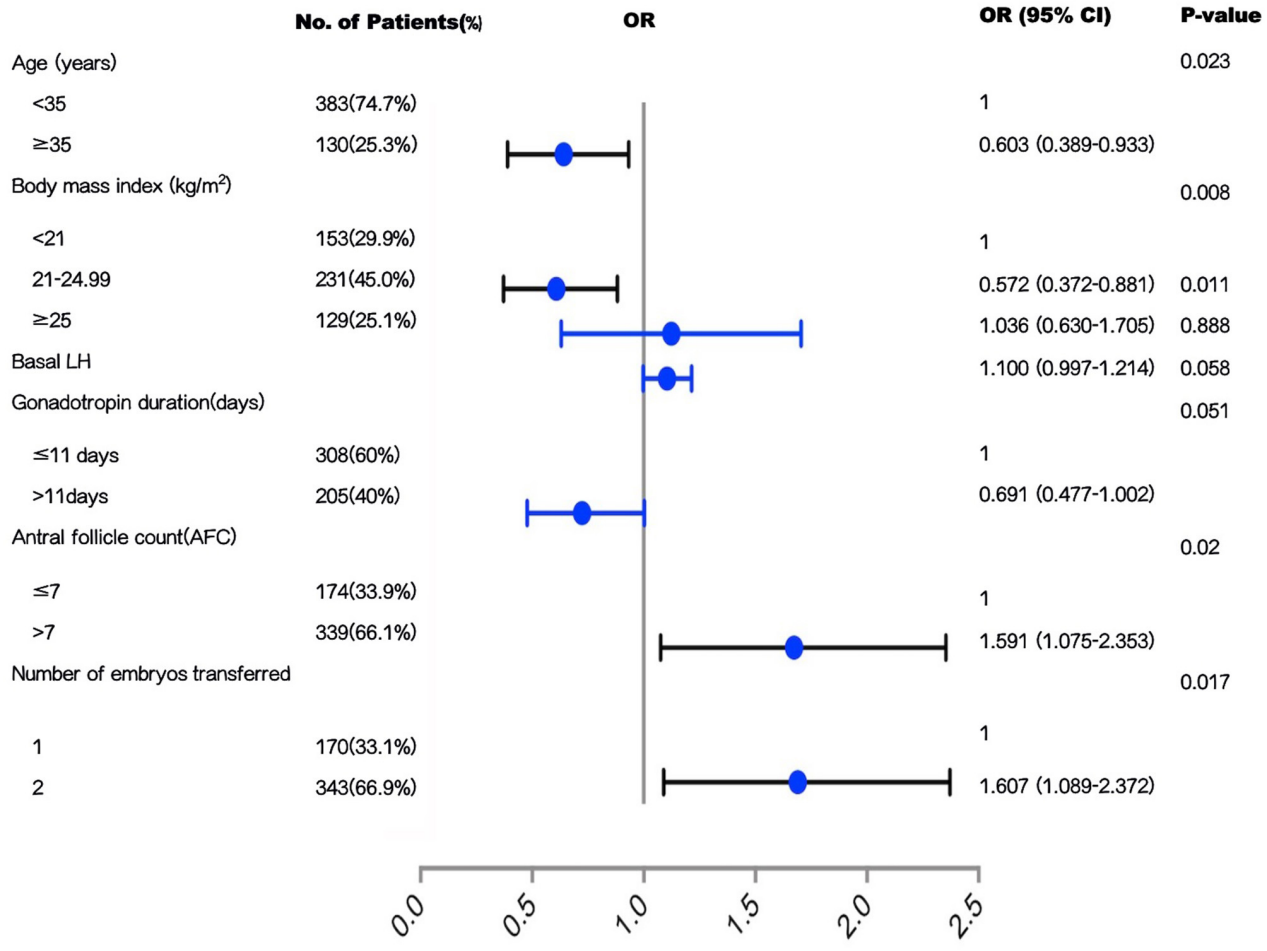
Forest plot to show the results of multivariate analysis.

We performed univariate regression analysis of variables predicting the incidence of live birth, as shown in Table 3. On the basis of univariate analysis results, female age, BMI, basal LH, ovarian stimulation protocol, start-up gonadotropin dosage, antral follicle counts, stage of embryo transferred, number of embryos transferred were included in the multivariable analysis, as shown in Table 4. The Hosmer-Lemer goodness-of-fit test showed *P* = 0.177, which indicated that the data fitted the model well. Female age, BMI, basal LH, AFC, ovarian stimulation protocol, start-up gonadotropin dosage, gonadotropin duration, stage of embryos transferred, number of embryos transferred were included in the logistic regression model. Multivariable modified Poisson regression models showed that two factors were significantly lowers the probability of live birth: female age ≥ 35 years (aOR 0.603; 95% CI 0.389–0.933; *P* = 0.023); BMI range 21–24.99 kg/m^2^ compared with BMI <21 kg/m^2^ (aOR 0.572; 95% CI 0.372–0.881, *P* = 0.011). And two factors significantly increased the probability of live birth: AFC >7 (aOR 1.591; 95% CI 1.075–2.353; *P* = 0.020); two embryos transferred (aOR 1.607; 95% CI 1.089–2.372; *P* = 0.017). The area under the curve (AUC) of the receiver operating characteristic (ROC) curve for the live birth rate prediction model was 0.757 (95% CI 0.610–0.740) indicating a fair performance. The forest plot shown in Figure 1 presents the results of our multivariate analysis.

**Table 3:** Univariate regression analysis of variables predicting the incidence of live birth in fresh IVF/ICSI-ET cycles.

**Predictor variable**	**Univariate analysis**	
	**OR (95% CI)**	***P*-value**
Age (years)		0.005
<35	1	
≥35	0.557 (0.370–0.840)	
Body mass index (kg/m^2^)		0.003
<21	1	
21–24.99	0.529 (0.349–0.800)	0.003
≥25	0.940 (0.588–1.504)	0.797
Type of infertility		0.944
Primary infertility	1	
Secondary infertility	0.988 (0.696–1.402)	
Surgical side of endometrioma cystectomy		0.545
Unilateral endometrioma cystectomy	1	0.281
Bilateral endometrioma cystectomy	0.892 (0.615–1.293)	
Time of surgeries		0.415
Primary surgery	1	
Recurrent surgery	0.723 (0.332–1.577)	
Interval between cystectomy and embryo transfer (month)	0.997 (0.991–1.003)	0.259
Duration of infertility (years)	0.997 (0.932–1.066)	0.919
Basal FSH (IU/L)	0.979 (0.917–1.045)	0.528
Basal LH (IU/L)	1.090 (0.995–1.195)	0.064
Basal E2 (pg/ml)	0.999 (0.992–1.006)	0.780
Ovarian stimulation protocol		0.013
GnRH agonist long protocol	1	
GnRH agonist prolonged protocol	0.773 (0.453–1.184)	0.204
GnRH agonist short protocol	0.504 (0.330–0.769)	0.002
GnRH antagonist protocol	0.572 (0.308–1.061)	0.076
Oocyte fertilization method		0.140
IVF	1	
ICSI	1.469 (0.882–2.445)	
Gonadotropin duration (days)		0.029
≤ 11	1	
>11	1.485 (1.041–2.117)	
Start-up gonadotropin dosage (IU)		0.029
<200	1	
≥200	0.679 (0.479-0.962)	
Total gonadotropin dosage(IU)-mean ± SD		0.537
<2,000	1	
≥2,000	1.116 (0.778–1.581)	
Endometrial thickness on HCG trigger day (mm)		0.065
<1.1 cm	1	
≥1.1 cm	1.402 (0.979–2.008)	
Ostradiol level on HCG trigger day; (pg/ml)		0.187
<2,000; *n* (%)	1	
2,000–3,000; *n* (%)	0.698 (0.458–1.066)	0.096
≥3,000; *n* (%)	0.994 (0.655–1.510)	0.978
Antral follicle count(AFC)		0.001
≤ 7	1	
>7	1.726 (1.118–2.506)	0.004
Number of oocytes ≥14 mm on HCG trigger day	1.000 (0.955–1.047)	1.000
Number of oocytes obtained	1.024 (0.981–1.068)	0.281
Stage of embryo transferred		0.033
Cleavage transfer	1	
Blastocyst transfer	0.632 (0.415–0.963)	
Number of top quality embryos transferred		0.219
0	1	
1	0.761 (0.464–1.249)	0.280
2	1.242 (0.826–1.867)	0.298
Number of embryos transferred		0.004
1	1	
2	1.746 (1.199–2.541)	

*FSH, follicle stimulating hormone; LH, luteinizing hormone; E2 estradiol; GnRH, gonadotrophin-releasing hormone; HCG, human chorionic gonadotropin*.

**Table 4:** Multivariate regression analysis of variables predicting the incidence of live birth in fresh IVF/ICSI-ET cycles.

**Predictor variable**	**Multivariate analysis**	
	**OR (95% CI)**	***P*-value**
Age (years)		0.023
<35	1	
≥35	0.603 (0.389–0.933)	
Body mass index (kg/m^2^)		0.008
<21	1	
21–24.99	0.572 (0.372–0.881)	0.011
≥25	1.036 (0.630–1.705)	0.888
Basal LH	1.100 (0.997–1.214)	0.058
Gonadotropin duration (days)		0.051
≤ 11 days	1	
>11days	0.691 (0.477–1.002)	
Antral follicle count (AFC)		0.020
≤ 7	1	
>7	1.591 (1.075–2.353)	
Number of embryos transferred		0.017
1	1	
2	1.607 (1.089–2.372)	

*LH, luteinizing hormone*.

## Discussion

The main objective of our study was to assess the factors associated with live birth rate in infertile women who underwent their first IVF/ICSI–ET after cystectomy for ovarian endometriosis. In this single-center case-control study of the records of 513 patients, we found that female age, BMI, AFC, and number of embryos transferred were important factors that affected the live birth rate. When age was considered as an independent variable, the results showed that older patients (35 years old) had a lower live birth rate (23.8%). Multivariate analysis confirmed these findings and identified patient age as a factor independently related to live birth rates. Such conclusions are generally regarded as dogma by IVF procedures (26–28), seem to be also applicable in patients following ovarian cystectomy for endometriomas.

Here we found that the relationship between BMI and the live birth rate was quadratic (a *U*-shaped curve), rather than linear (a straight line). After multivariable analysis, the live birth rate was higher for women with a BMI ≤ 21 kg/m^2^ and BMI > 25 kg/m^2^ than for those in the BMI range of 21–24.99 kg/m^2^. This means that when we considered the impact of BMI on reproductive outcomes, we can not only divide them into groups according to WHO criteria, but also divide each group into subgroups for analysis. Previous research on relatively large cohorts also showed that plots of female BMI against ART outcome showed an inverted *U* or *J* shape. Fedorcsák et al. found that increased BMI in women receiving IVF or ICSI was associated with a lower live birth rate and a higher incidence of early miscarriage (29). Wang et al. showed that compared with the reference group (BMI, 18.5–24.9 kg/m^2^), thin women had a similar risk of spontaneous abortion, whereas there was progressive increase in risk among overweight, obese, and very obese groups (30). Veleva et al. found that obese and underweight women had an increased risk of miscarriage (31). Bellver et al. (32) and Dechaud et al. (33) both divided woman into 4 groups:lean (<20 kg/m^2^; *n* 1/4 1,070; 16.5%); normal (20–24.9 kg/m^2^; *n* 1/4 3,930; 60.5%); overweight (25–29.9 kg/m^2^; *n* 1/4 1,081; 16.6%); and obese (R30 kg/m^2^; *n* 1/4 419; 6.4%). Bellver et al. concluded that the cumulative pregnancy rate after four IVF cycles was reduced as BMI increased. Rittenberg et al. suggest that raised BMI is associated with adverse pregnancy outcome in women undergoing IVF/ICSI treatment, including lower live birth rate which was partially consistent with our study (34). However, several other studies did not find significant differences in clinical pregnancy or live birth rates between different BMI groups (35, 36).

Ovarian reserve is defined as the functional potential of the ovary, which reflects the number and quality of remaining follicles. By optimizing the controlled ovarian stimulation (COS) program, increasing the proportion of normal responders can increase the live birth rate. Assessing ovarian reserve may be helpful in predicting adverse reactions to ovarian hyperstimulation for IVF, and both AFC and serum anti-Müllerian hormone (AMH) levels are indicators that accurately predict ovarian response (37). Moreover, the serum AMH concentration might not accurately assess the response of the ovaries of women with endometrioma to COS. However, AFC appears to be a more reliable marker of ovarian reserve in patients with ovarian endometrioma (38). In 2011, the European Society for Human Reproduction and Embryology (ESHRE) defined the abnormal ovarian reserve as being an AFC <5–7 follicles or AMH <0.5–1.1 ng/mL. The AFC has predictive value for ovarian response in IVF cycles, with a cut-off value of 7 follicles above which there are more chances of normal response (39). A Dutch prospective cohort study performed an open-label multicenter randomized controlled trial (RCT) in women with a predicted poor ovarian response (AFC <11) undergoing IVF/ICSI. However, an increased dose of FSH did not improve cumulative live birth rates compared with a standard dose (40). Li et al. found that the AFC was an independent factor associated with cumulative clinical pregnancy rates in a population with endometriosis and a control group (23). Here, the live birth rate was significantly lower in the group of women with an AFC of ≤ 7 group than in those with an AFC > 7. Our multivariate analysis confirmed AFC as a factor independently associated with the live birth rate. From a practical point of view in the ART clinic, the best cut-off value to achieve a higher live birth rate for patients after cystectomy for endometrioma was an AFC of > 7.

According to our results, the number of fresh embryos transferred was an important clinical factor influencing the live birth rate; that associated with the transfer of two fresh embryos was higher than that associated with the transfer of one embryo. A meta-analysis indicated that double-embryo transfer presented a significantly higher live birth rate than single-embryo transfer (41). In recent years, multiple ET during IVF cycles increases the multiple pregnancy rates leading to increased maternal and perinatal morbidity rates (42). Therefore, a single ET is now being seriously considered as a means to reduce the risk of multiple pregnancy. However, this needs to be weighed against the risk of endangering the overall live birth rate.

In practical ART clinic work, how to choose a suitable ovarian stimulation protocol for patients with endometriosis is still controversial. In 2010, Sallam et al. published a study in the Cochrane Database, which combined the results of three prospective clinical studies and concluded that 3–6 months of pre-treatment with a GnRH agonist could increase the IVF pregnancy rate of patients with endometriosis by 4-fold and increase the IVF live birth rate of patients with endometriosis by 9-fold (43). In 2020, an RCT was conducted to evaluate the effect of GnRH agonist treatment for 3 months before IVF on the clinical pregnancy rate of patients with infertile endometriosis compared with placebo. However, there were no significant differences in implantation or clinical pregnancy rate between the 2 groups, and the cumulative live birth rate was not significantly different between the 2 groups (44). Many studies have included only 2 or 3 COS protocols to compare reproductive outcomes. However, here we included four COS regimens: a long GnRH agonist protocol; a prolonged GnRH agonist protocol; a GnRH antagonist protocol; and a short GnRH agonist protocol. Univariate regression analysis of the data showed that the live birth rate of the short GnRH agonist protocol was statistically lower than that of the long GnRH agonist protocol. There was no statistically significant difference in the live birth rate between the long GnRH protocol and the antagonist protocol. When using adjusted multivariate regression analysis, the COS protocols were not independent factors in influencing the live birth rate. We consider that COS protocols might affect the number and quality of oocytes, but does not affect fetal growth after successful ET.

Our study had some limitations. First, it was a retrospective study, and medical records might be limited and selection bias existed. Second, the sample size was relatively small and could not exclude all potential biases. Our conclusions need to be tested further in prospective, large-scale multicenter clinical trials.

## Data Availability Statement

The raw data supporting the conclusions of this article will be made available by the authors, without undue reservation.

## Ethics Statement

The studies involving human participants were reviewed and approved by Institutional Review Board of the School of Medicine, Shandong University (No. 2019-1-14). Written informed consent for participation was not required for this study in accordance with the national legislation and the institutional requirements.

## Author Contributions

JM and LY contributed to conception and design of the study. WL, TS, and YH organized the database. WL and ZG performed the statistical analysis. WL wrote the first draft of the manuscript. LY, ZG, TS, and YH wrote sections of the manuscript. All authors contributed to manuscript revision, read, and approved the submitted version.

## Conflict of Interest

The authors declare that the research was conducted in the absence of any commercial or financial relationships that could be construed as a potential conflict of interest.
